# Blood flow restriction training: a new approach for preventing and treating sarcopenia in older adults

**DOI:** 10.3389/fphys.2025.1616874

**Published:** 2025-08-25

**Authors:** Wei Li, Mingzhen Hu, Qiushi Yin, Yuqing Liu, Lin Chen, Qin Ru, Guodong Xu, Yuxiang Wu

**Affiliations:** ^1^ Department of Sports Medicine, Wuhan Sports University, Wuhan, China; ^2^ Institute of Intelligent Sport and Proactive Health, Department of Health and Physical Education, Jianghan University, Wuhan, China

**Keywords:** sarcopenia, aging, blood flow restriction training, muscle mass, muscle strength

## Abstract

With the intensification of population aging, sarcopenia in older adults has become a significant public health issue affecting quality of life. Sarcopenia is a progressive and systemic skeletal muscle disorder characterized by reduced muscle mass, decreased muscle strength, and diminished physical function. Although conventional exercise interventions have shown some efficacy in managing sarcopenia, their effects are limited and often insufficient to effectively halt disease progression. Therefore, exploring more efficient exercise interventions is of great importance. Blood flow restriction training (BFRT), as an emerging exercise intervention, has garnered increasing attention in recent years for its application in sarcopenia among older adults. Studies suggest that, compared to traditional resistance exercise, BFRT demonstrates superior effectiveness in improving muscle strength and mass in older adults, potentially serving as a viable alternative to conventional training methods. However, BFRT also presents certain limitations, including potential risks such as cardiovascular responses and muscle injury. Therefore, careful consideration of appropriate application scenarios and exercise loads is crucial during its implementation. This study reviews the biological mechanisms of BFRT in the intervention of sarcopenia and proposes tailored training protocols and application models for older adults. Furthermore, it thoroughly examines the potential risks and applicability of BFRT, aiming to provide theoretical foundations and practical guidance for clinical application. Additionally, the limitations of current research are analyzed, offering recommendations for future research directions.

## 1 Introduction

Skeletal muscle, one of the largest organs in the human body, accounts for approximately 40% of total body weight and plays a crucial role in controlling daily movement, thermoregulation, physical strength, and metabolic homeostasis (Yadav et al., 2022; Jing et al., 2022). Maintaining skeletal muscle health—including muscle strength, muscle mass, and muscle function (Cawthon et al., 2022)—is a critical prerequisite for extending healthy lifespan (McLeod et al., 2016; Tieland et al., 2018).

However, with advancing age, skeletal muscle strength and mass progressively decline at annual rates of 2%–3% and 0.5%–1% respectively (Goodpaster et al., 2006). This pathological condition is formally defined as sarcopenia—a geriatric syndrome characterized by progressive, generalized loss of skeletal muscle mass, strength, and physical function (Cruz-Jentoft and Sayer, 2019). The European Working Group on Sarcopenia in Older People (EWGSOP2) specifically emphasized in 2018 that reduced muscle strength should serve as the primary diagnostic criterion (Cruz-Jentoft et al., 2019). Etiologically, sarcopenia can be classified into two categories: primary (age-related) and secondary (associated with comorbidities such as rheumatoid arthritis, systemic sclerosis, and osteoarthritis) (Cruz-Jentoft et al., 2019; Bauer et al., 2019). Sarcopenia impairs physical function and increases risks of falls, disability, and mortality, severely impacting elderly health and quality of life (Evans et al., 2024). It also raises healthcare costs, creating substantial individual and societal burdens (Tournadre et al., 2019). Effective interventions are therefore essential to manage this age-related condition and improve health outcomes (Scott et al., 2021).

Currently, resistance training is widely recognized as the most effective intervention for sarcopenia (Dent et al., 2018; Fragala et al., 2019). However, its application may be limited due to its intensity characteristics. High-load resistance training (>70% 1RM) may increase the risk of exercise-related injuries due to its high mechanical loading properties (Schutzer, 2004). Furthermore, this training modality often results in reduced compliance and adherence among older adults with comorbidities (e.g., frailty, sarcopenia), rehabilitation patients, and individuals experiencing acute pain episodes (Fragala et al., 2019; Lavallee and Balam, 2010; Lees et al., 2005). Therefore, exploring safe, efficient, and low-load alternative interventions is urgently needed.

In recent years, blood flow restriction training (BFRT) has garnered significant attention as an emerging therapeutic intervention (Slysz et al., 2016). BFRT involves applying external pressure to the proximal limb using compression devices (e.g., tourniquets or inflatable cuffs) during exercise, partially restricting arterial inflow and fully occluding venous outflow. This creates an ischemic and hypoxic environment within the muscle tissue (Beckwée et al., 2019), triggering a cascade of physiological processes related to tissue adaptation. BFRT affects skeletal muscle primarily by promoting the secretion of anabolic hormones, protein synthesis, recruitment of type II muscle fibers, cellular swelling, and the generation of reactive oxygen species and their derivatives [e.g., nitric oxide (NO) and heat shock proteins (HSPs)] (Yuan et al., 2023).

BFRT significantly enhances muscle strength and physical function in elderly populations by inducing muscle hypertrophy (Baker et al., 2020). Multiple studies confirm that BFRT achieves comparable outcomes to conventional resistance training for sarcopenia management in older adults (Fabero-Garrido et al., 2022; Kong et al., 2023; Mallmann et al., 2024), with superior efficacy particularly observed in strength improvement (Kong et al., 2023). Consequently, for elderly patients with comorbidities such as degenerative joint disorders or cardiovascular diseases, BFRT emerges as a viable alternative intervention that simultaneously mitigates injury risks associated with high-load training while achieving equivalent strength gains (Patterson et al., 2019; Hughes et al., 2017; Ladlow et al., 2018; Zhang T. et al., 2022; Mallmann et al., 2024).

However, existing research still shows inconsistencies or requires further exploration in several key areas: the biological mechanisms, optimal training protocols (including resistance, cuff width, load, repetitions, sets, frequency, etc.), application effects (such as BFR applied alone or combined with aerobic exercise or resistance training), and safety concerns. Therefore, the aim of this review is to systematically integrate existing evidence, with a focus on elucidating the biological mechanisms of BFRT for sarcopenia intervention in older adults. Based on these mechanisms, we will propose tailored training protocols and application models suitable for elderly sarcopenia patients. Furthermore, we will comprehensively evaluate the safety profiles and potential risks of BFRT, thereby providing an evidence-based foundation for its long-term clinical application and broader implementation in geriatric sarcopenia management.

## 2 Biological mechanisms of BFRT intervention in sarcopenia

**FIGURE 1:**
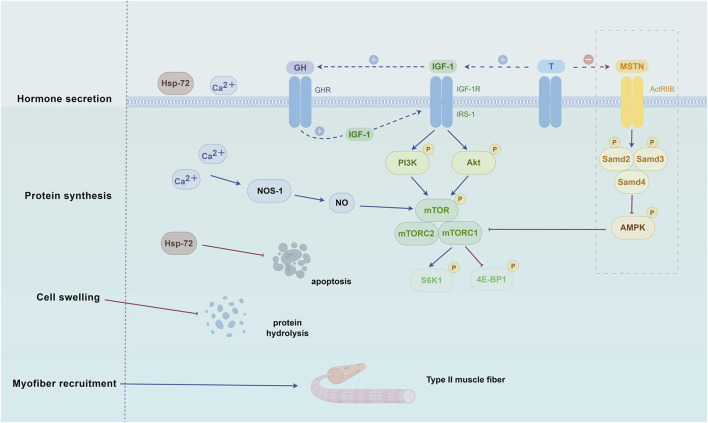
Biological Mechanisms of BFRT Intervention in Sarcopenia. The mechanisms by which BFRT addresses sarcopenia in the elderly primarily include promoting the secretion of anabolic hormones, regulating protein synthesis, accelerating the recruitment of type II muscle fibers, inducing cellular swelling, and stimulating the production of reactive oxygen species and their derivatives (e.g., NO and HSPs). The illustration was created with figdraw (figdraw.com). Abbreviations: 4E-BP1, eukaryotic initiation factor 4E-binding protein 1; ActRIIB, activin receptor type-2B; Akt, protein kinase B; HSP-72, heat shock protein-72; IGF-1, insulin-like growth factor-1; IGF-1R, insulin-like growth factor 1 receptor; IRS-1, insulin receptor substrate-1; MSTN, myostatin; mTOR, mammalian target of rapamycin; NOS-1, nitric oxide synthase-1; PI3K, phosphatidylinositol 3-kinase; S6K1, ribosomal protein S6 kinase 1; T, testosterone.

The pathogenesis of sarcopenia involves multiple factors, including nutritional deficiencies, chronic inflammation, hypogonadism, abnormal myokine secretion, hormonal alterations, insulin resistance, motor neuron loss, impaired neuromuscular junction function, inadequate muscle blood flow, mitochondrial dysfunction, and satellite cell senescence (Zhang X. et al., 2022). Numerous studies have demonstrated that BFRT can induce muscle hypertrophy, thereby enhancing muscle strength and improving physical function in older adults (Baker et al., 2020; Fabero-Garrido et al., 2022; Kong et al., 2023; Mallmann et al., 2024). However, the specific mechanisms underlying these effects remain incompletely understood. In general, the mechanisms by which BFRT addresses sarcopenia in the elderly primarily include promoting the secretion of anabolic hormones, regulating protein synthesis, accelerating the recruitment of type II muscle fibers, inducing cellular swelling, and stimulating the production of reactive oxygen species and their derivatives (e.g., NO and HSPs) (Yuan et al., 2023). A deeper understanding of the biological mechanisms of BFRT in treating sarcopenia is essential for developing physiologically informed, personalized training protocols for elderly patients. The following sections will elaborate on these mechanisms, as illustrated in Figure 1.

### 2.1 BFRT promotes the secretion of anabolic hormones

#### 2.1.1 Growth hormone

Growth hormone (GH) and insulin-like growth factor-1 (IGF-1) play critical roles in maintaining skeletal muscle strength and mass. In the circulatory system, the secretion of GH stimulates the release of IGF-1, which in turn promotes GH secretion (Le Roith et al., 2001). Both hormones activate protein synthesis and inhibit protein degradation through the phosphatidylinositol 3-kinase/protein kinase B (PI3K/Akt) signaling pathway, thereby influencing muscle mass (Bian et al., 2020).

GH is a peptide hormone with multiple functions, including maintaining cardiac function, glucose homeostasis, bone mineralization, the balance of lipogenesis and lipolysis, and skeletal muscle anabolism. With aging, GH secretion gradually declines (Hage and Salvatori, 2023), and its deficiency leads to reduced muscle strength and mass. Research suggests that BFRT can increase GH secretion and potentially improve body composition and muscle performance. For instance, the study by Shimizu et al. (2016) demonstrated that in 040 older adults who underwent 4 weeks of BFRT (20% 1RM), GH levels significantly increased (from 0.9 ± 0.7 ng/mL to 3.1 ± 1.3 ng/mL, p < 0.05). However, since the participants were healthy older adults, the findings have limited applicability to elderly patients with sarcopenia. Additionally, compared to younger males, older males exhibit a blunted GH response following BFRT (p = 0.02) (Manini et al., 2012), suggesting that age may be a critical factor influencing the incidence and extent of muscle hypertrophy induced by BFRT.

#### 2.1.2 Insulin-like growth factor-1

In males aged 60 and above, the secretion of IGF-1 decreases with age, which is closely associated with the development of sarcopenia and muscle weakness (Chen et al., 2017). Research indicates that during aging (Musarò et al., 2001) and in animal models of neuromuscular diseases (Bosch-Marcé et al., 2011), the overexpression of IGF-1 in skeletal muscle can regulate protein synthesis and promote body growth. When IGF-1 binds to its receptor (insulin-like growth factor 1 receptor, IGF-1R), IGF-1R phosphorylates the intracellular adaptor protein insulin receptor substrate-1 (IRS-1), which then recruits and phosphorylates PI3K, subsequently activating Akt (Yoshida and Delafontaine, 2020). IGF-1 induces protein synthesis and muscle hypertrophy through the PI3K/Akt pathway, and the activation of Akt increases fiber size in the muscles of regenerating and adult rats while preventing denervation atrophy (Pallafacchina et al., 2002). In a case report, a 91-year-old male patient with sarcopenia showed a significant increase in plasma IGF-1 concentration after 3 months of low-intensity BFRT (30% 1RM) (Grutter Lopes et al., 2019). Additionally, Barjaste et al. (2021) found that a single session of BFR walking training led to an increase in IGF-1 levels. These findings suggest that BFRT enhances IGF-1 secretion, which may promote muscle hypertrophy and offer potential therapeutic benefits for sarcopenia.

However, a self-controlled trial by Ozaki et al. (2014) found that a single session of BFR walking exercise with a pressure of 240 mmHg and an intensity of 50% maximal oxygen uptake (VO_2_max) did not induce changes in IGF-1 levels. This contrasts with findings from (Barjaste et al., 2021) in a pre-post controlled study, which reported significant IGF-1 elevation after bilateral BFR training. The discrepancy may be attributed to differences in experimental design, Barjaste et al. applied bilateral BFR (i.e., restricting blood flow to both legs simultaneously), whereas Ozaki et al. used unilateral BFR (i.e., restricting blood flow to only one leg, with the other serving as a control). Unilateral BFR may not have generated sufficient metabolic stress or localized hypoxia to significantly stimulate IGF-1 secretion. Furthermore, a subsequent randomized controlled trial by Ozaki et al. (2017) demonstrated that changes in anabolic hormones (such as GH, insulin, and norepinephrine) were not significantly correlated with muscle hypertrophy induced by BFR walking. These preliminary results suggest that the elevation of anabolic hormones induced by BFR walking may have limited impact on muscle growth. In this regard, animal studies have indicated that when other factors, such as myostatin, heat shock protein-72 (HSP-72), and nitric oxide synthase-1 (NOS-1), undergo changes favorable to muscle growth, IGF-1 may not be essential for muscle hypertrophy (Kawada and Ishii, 2005).

#### 2.1.3 Testosterone

Testosterone is one of the most important sex hormones in plasma, playing a critical role in protein, carbohydrate, and fat metabolism. It is essential for maintaining muscle mass and function, bone mass, and body composition (MacLean et al., 2008). After the age of 30, blood levels of testosterone decline by 1%–2% annually (Tajar et al., 2010), accompanied by a reduction in muscle mass, strength, and physical function (Perry et al., 2000). Therefore, testosterone is considered a key factor in preserving muscle mass and function during aging (Galvão et al., 2008).

The positive effects of testosterone on skeletal muscle involve several mechanisms. First, testosterone-induced increases in muscle volume are associated with concentration-dependent increases cross-sectional areas (CSA) of both type I and type II muscle fibers and myonuclear number (Sinha-Hikim et al., 2002). Second, testosterone promotes the activation and proliferation of satellite cells, enhancing muscle regeneration in both young and aged mice (Serra et al., 2013). Additionally, testosterone upregulates IGF-1 expression (Ferrando et al., 2002) and downregulates the gene expression of myostatin (Dubois et al., 2014). Studies have shown that low-intensity BFRT effectively increases levels of GH, IGF-1, and testosterone in young men, thereby enhancing muscle anabolic potential and promoting hormone secretion (Yinghao et al., 2021). However, since these studies focused solely on young individuals, their findings have limited applicability to older adults. In conclusion, while testosterone secretion can effectively improve muscle mass and strength in elderly patients, its specific effects on muscle function remain unclear. Therefore, testosterone cannot be considered a unique mechanism through which BFRT mitigates sarcopenia.

In summary, BFRT may improve sarcopenia by modulating hormone levels during exercise. However, it should be noted that current evidence for BFRT-induced hormonal responses (e.g., GH, IGF-1, testosterone) in elderly populations with sarcopenia remains limited, with most studies conducted in healthy older adults or younger individuals. Furthermore, Laurentino et al. (2022) found that although BFRT significantly increased muscle mass and strength, along with elevated levels of GH, IGF-1, and testosterone, statistical analysis revealed no significant correlation between these improvements and hormonal changes (p > 0.05). This implies that the primary mechanisms of BFRT may operate independently of systemic hormonal regulation, particularly in the understudied sarcopenic population. Further studies targeting hormonal pathways in sarcopenia cohorts are needed to clarify their precise role.

### 2.2 BFRT regulates protein synthesis

Muscle growth primarily occurs through enhancing anabolic signals or inhibiting catabolic pathways to promote protein synthesis. Among the key mechanisms regulating muscle mass and protein synthesis, the mammalian target of rapamycin (mTOR) signaling pathway plays a central role (Mukund and Subramaniam, 2020). As a highly conserved serine/threonine protein kinase, mTOR is crucial for skeletal muscle hypertrophy. mTOR exists in two distinct functional complexes: mTORC1 and mTORC2 (Marcotte et al., 2015).

Specifically, mTORC1 is pivotal in regulating protein synthesis and muscle mass. Research indicates that mTORC1 promotes protein synthesis and skeletal muscle hypertrophy by activating its downstream target ribosomal protein S6 kinase 1 (S6K1) and inhibiting eukaryotic initiation factor 4E-binding protein 1 (4E-BP1) (Goodman, 2013). Furthermore, mTOR serves as a critical node in the protein synthesis signaling pathway, with its activity regulated by various upstream signaling molecules. For example, IGF-1 activates mTOR through the PI3K/Akt pathway, thereby inducing skeletal muscle hypertrophy (Barbé et al., 2015). Consequently, mTOR acts as a bridge in the regulation of skeletal muscle hypertrophy, capable of both receiving upstream signals and influencing downstream pathways to comprehensively participate in the regulation of muscle hypertrophy. Additionally, the upregulation of reactive oxygen species and their derivatives (e.g., NO and HSPs) and the downregulation of myostatin may also positively influence protein synthesis.

#### 2.2.1 BFRT promotes protein synthesis

BFRT enhances mTOR signaling through activation of the PI3K/Akt pathway (Mukund and Subramaniam, 2020), thereby stimulating muscle protein synthesis and inducing skeletal muscle hypertrophy. In elderly populations prone to sarcopenia, low-intensity BFRT has been shown to effectively activate the mTORC1 signaling pathway and promote muscle protein synthesis. A study by Fry et al. (2010) demonstrated that in older adults, muscle protein synthesis increased significantly by 56% at 3 h post-BFRT exercise, accompanied by a notable rise in the phosphorylation levels of S6K1 and Akt. This indicates enhanced mTORC1 signaling following BFRT. This indicates enhanced mTORC1 signaling following BFRT. The strengthening of mTORC1 signaling suggests an enhancement in translation initiation, which may explain how BFRT promotes muscle protein synthesis. Moreover, S6K1 phosphorylation, a key regulator of exercise-induced muscle protein synthesis, has been shown to increase with BFRT (Loenneke et al., 2010). Fujita’s research revealed that in young men, S6K1 phosphorylation levels were significantly higher in the BFRT group (20% 1RM, 200 mmHg) compared to the non-BFR control group at 3 h post-exercise, with a 46% increase in muscle protein synthesis (Fujita et al., 2007). However, these findings are limited to young males, necessitating further research to elucidate the specific biological mechanisms by which BFRT regulates protein synthesis. The phosphorylation of the 4E-BP1 protein is also closely associated with the mTORC1 signaling pathway (Böhm et al., 2021). Research by Le Bacquer et al. (2019) demonstrated that knockout of the 4E-BP1 gene enhanced muscle protein synthesis in mice, accompanied by increased muscle strength (assessed via standardized grip test) and muscle mass (P < 0.05). These findings suggest that 4E-BP1 deficiency may improve muscle mass and function in aging mice, potentially by mitigating energy metabolism disorders. This implies that 4E-BP1 phosphorylation could be a therapeutic target for sarcopenia. BFRT, by inhibiting 4E-BP1 phosphorylation, may play a positive role in mitigating sarcopenia.

Research has also shown that during BFRT, elevated intracellular Ca^2+^ concentrations or blood flow reperfusion can activate neuronal nitric oxide synthase (NOS) and produce NO (Howard et al., 1995). NO production is associated with mTOR activation and linked to protein synthesis (Ito et al., 2013). Furthermore, NO mediates the activation of satellite cells (Anderson, 2000). Previous research has observed a significant increase in NOS expression following BFRT (Larkin et al., 2012). Therefore, NO may play a crucial role in the muscle adaptations induced by BFRT.

HSPs are induced under hypoxic, ischemic reperfusion, and acidotic conditions and function as chaperones to prevent misfolding or aggregation of proteins under metabolic stress caused by compression training (Loenneke et al., 2010). Research has shown that HSP-72 not only protects against muscle protein loss but also mitigates the decline in protein synthesis caused by disuse muscle atrophy and inhibits apoptosis, thereby reducing muscle mass loss (Naito et al., 2000). Kawada and Ishii were the first to report a significant increase in HSP-72 in the plantar muscles of rats after 2 weeks of BFRT (Kawada and Ishii, 2005). These findings are associated with significant muscle hypertrophy, suggesting that the upregulation of HSP-72 may be a potential mechanism by which BFRT promotes skeletal muscle hypertrophy and attenuates atrophy. However, Fry et al. found no significant increase in Heat shock protein-70 (HSP-70) levels after BFRT (20% 1RM, 200 mmHg) (Fry et al., 2010). These conflicting data may indicate that only specific HSPs, such as HSP-72, play a role in muscle hypertrophy, while others, like HSP-70, do not exhibit similar effects. To better understand the mechanisms of BFRT-induced muscle hypertrophy, further research is needed to investigate different heat shock protein subtypes and identify those with significant post-exercise anabolic roles.

In summary, the activation of the mTOR signaling pathway, increased levels of NOS, and elevated expression of HSP-72 following BFRT may serve as key mechanisms that contribute to the enhanced muscle protein synthesis and subsequent skeletal muscle hypertrophy induced by BFRT.

#### 2.2.2 BFRT inhibits protein breakdown

In addition to promoting protein synthesis, BFRT also prevents skeletal muscle atrophy by inhibiting the expression of proteins associated with protein degradation. Myostatin, also known as growth differentiation factor 8, is primarily expressed in skeletal muscle (Baczek et al., 2020) and acts as a negative regulator of muscle mass and development (Guo et al., 2020). Myostatin binds to the activin receptor type-2B (ActRIIB), leading to the phosphorylation of Smad2 and Smad3, which then form a heterodimer with Smad4 and translocate to the nucleus to regulate gene transcription, thereby negatively regulating muscle growth. This process not only activates genes involved in muscle protein degradation but also suppresses protein synthesis by inhibiting the IGF-1/Akt/mTOR pathway (Bilski et al., 2022). Research has shown that myostatin levels increase with age and muscle mass loss (Yarasheski et al., 2002). For instance, patients with sarcopenia exhibit significantly elevated myostatin levels alongside reduced Akt phosphorylation efficiency. Specifically, myostatin mRNA and protein levels in sarcopenia patients increased by 2-fold and 1.4-fold, respectively, while Akt phosphorylation efficiency decreased by 30% (Léger et al., 2008). This suggests that elevated myostatin levels may be a key factor in the development of age-related sarcopenia. Therefore, inhibiting the expression of myostatin -related proteins is of significant importance for treating sarcopenia in the elderly.

Studies have also demonstrated that BFRT can reduce myostatin expression, thereby positively impacting the improvement of sarcopenia. For example, Laurentino et al. (2012) found that after 8 weeks of BFR knee extension training (20% 1RM), myostatin mRNA expression decreased by 45%. This indicates that low myostatin expression may play a role in the muscle hypertrophy induced by BFRT.

### 2.3 BFRT accelerates type II muscle fiber recruitment

Multiple studies have demonstrated significant type II muscle fiber atrophy in the skeletal muscles of older adults (Larsson, 1978; Verdijk et al., 2007; Martel et al., 2006; Snijders et al., 2009). Given that type II fast-twitch fibers predominantly contribute to high-intensity contractions, their atrophy may lead to decreased muscle strength in the elderly, ultimately promoting the development and progression of sarcopenia (Cruz-Jentoft et al., 2019). BFRT enhances type II fiber recruitment, representing a key mechanism for its muscle growth promotion. Research indicate that muscle hypertrophy induced by BFRT involves significant increases in CSA of both type I and II muscle fibers (Libardi et al., 2024; Wang et al., 2023). Loenneke et al. (2011a) confirmed that compared to conventional training, low-intensity BFRT significantly improves motor unit recruitment efficiency, particularly in activating type II fibers. In elderly populations, a 6-week BFRT intervention (30% 1RM) increased type I/II fiber CSA by approximately 20% (P < 0.05), while significantly enhancing maximal strength and endurance (Wang et al., 2023).

At the molecular level, BFRT-induced metabolic stress modulates calcium ion dynamics (release/reuptake), thereby strengthening actin-myosin interactions (Berchtold et al., 2000) and facilitating type II fiber activation. Given type II fibers’ fast-twitch properties and high-force output characteristics, their preferential recruitment enables greater force production under lower loads, which critically contributes to strength development and hypertrophy. These findings establish BFRT as an effective intervention for age-related declines in skeletal muscle mass and function (e.g., sarcopenia) (Conceição and Ugrinowitsch, 2019; Hughes et al., 2017). However, the cellular adaptation mechanisms to BFRT in aging individuals remain incompletely elucidated.

### 2.4 BFRT induces cell swelling

Current evidence suggests that BFRT-induced increases in muscle mass and strength may be associated with acute cell swelling mechanisms. Loenneke et al. proposed that BFR triggers cell swelling by increasing intracellular osmotic pressure, thereby activating the mTOR pathway to promote protein synthesis and muscle hypertrophy (Loenneke et al., 2012c). This hypothesis is supported by counterevidence: studies show that cellular dehydration downregulates mTOR signaling and impairs protein metabolism regulation (Schliess et al., 2006). However, it should be noted that this mechanism was initially based on hepatocyte research, and its translatability to human skeletal muscle remains unverified.

Recent experiments have challenged the swelling hypothesis: Nyakayiru et al. (2019) found that BFR alone (without muscle contraction) did not significantly enhance myofibrillar protein synthesis. This suggests that if cell swelling does exert anabolic effects, its mechanism may involve inhibiting protein breakdown (e.g., by downregulating MURF1 expression) (Kakehi et al., 2020). Alternatively, the study’s experimental conditions (resting state in healthy young males) may have limited observable effects, with BFR benefits potentially more pronounced under immobilization or other physiological stressors.

Furthermore, the relationship between BFR pressure gradients and the degree of cell swelling warrants investigation. Theoretically, moderate external pressure may induce cell swelling, activating mTOR and other anabolic pathways to promote protein accretion, whereas insufficient pressure may fail to provide adequate stimulus, and excessive pressure could cause ischemic injury or disrupt cell volume regulation, thereby diminishing efficacy (Loenneke et al., 2011b). However, direct evidence establishing a dose-response relationship between BFR pressure levels and cell swelling remains lacking, necessitating further mechanistic studies.

Despite extensive evidence of post-BFRT cell swelling, research on this mechanism remains limited, and no studies have confirmed whether swelling-induced suppression of catabolism contributes to muscle growth. Future studies should clarify the relationship between BFR intensity and cell swelling, and determine whether cellular edema plays a pivotal role in human muscle hypertrophy signaling. These findings would hold significant clinical implications for populations with exercise contraindications.

In summary, although multiple potential mechanisms of BFRT for intervening in age-related sarcopenia have been proposed, the mechanisms underlying BFRT-induced muscle adaptation remain incompletely understood. The primary mechanisms by which BFRT stimulates muscle growth include: 1) increasing the secretion of anabolic hormones (e.g., GH, IGF-1, and testosterone); 2) enhancing mTOR signaling through the PI3K/Akt pathway and suppressing the expression of proteins associated with protein degradation (e.g., myostatin), thereby promoting protein synthesis; 3) altering motor unit recruitment patterns to accelerate the recruitment of type II muscle fibers; 4) activating the mTOR pathway through induced cell swelling, promoting protein synthesis and muscle hypertrophy. Additionally, studies have shown that BFRT can influence the expression of HSP-72 and NOS-1. In conclusion, low-intensity BFRT appears to exert its effects through multiple mechanisms. However, research on these mechanisms remains incomplete, and further studies are needed to clarify the specific mechanisms by which BFRT intervenes in sarcopenia and to determine the impact of related factors on muscle activation.

## 3 Training protocols for BFRT intervention in sarcopenia

The efficacy of BFRT is influenced by various training parameters, making the development of scientifically sound training protocols crucial for the prevention and treatment of sarcopenia in older adults. However, several challenges and research limitations persist in practical applications. For instance, there is a lack of consistency in the applied restrictive pressure across studies, and research on BFRT in older adults with sarcopenia remains limited. Therefore, based on existing literature, this section discusses the impact of six key factors—cuff type and width, restrictive pressure, intermittent *versus* continuous application, training load and intensity, training frequency, and inter-set rest intervals and training volume—on the effectiveness of BFRT in sarcopenia intervention. Additionally, recommendations for BFRT training protocols are proposed (see Table 1).

**TABLE 1:** Factors affecting BFRT efficacy in sarcopenia and recommendations.

Factor	Recommended training protocol	Precautions	References
Cuff Type	Use inflatable cuffs for BFR to allow precise pressure control	No significant difference between materials; similar BFR effect	Yasuda et al. (2012)
Cuff Width	Upper limb: 5–9 cm; Lower limb: 10–15 cm	Ensure cuff width matches limb circumference; avoid excessive width affecting target muscle groups	Patterson et al. (2019), Mallmann et al. (2024)
Restrictive Pressure	40%–80% AOP.	Avoid excessive pressure to prevent discomfort or safety risks; monitor pressure regularly	Patterson et al. (2019)
Intermittent and Continuous BFR	Use intermittent BFR mode	Observe patient response during pressure release; ensure adequate blood flow recovery	Abe et al. (2006), Schwiete et al. (2021)
Training Load and Intensity	20%–40% 1RM/MVC or RPE 6–8/10	Avoid excessive load to reduce joint stress and injury risk; adjust intensity based on tolerance	Patterson and Ferguson (2011), Karabulut et al. (2010), Kong et al. (2023)
Training Frequency and Rest Intervals	2–3 sessions per week; Rest intervals of 30–60 s between sets	Adjust rest intervals based on fatigue; avoid excessive rest to maintain metabolic stress	Kong et al. (2023)
Training Volume	Perform 3–4 sets per session; 15–30 repetitions per set.	Gradually increase volume based on patient tolerance	Loenneke et al. (2012d), Abe et al. (2005), Patterson and Ferguson (2011), Clarkson et al. (2017), Cook et al. (2018)

### 3.1 Cuff type and width

The selection of cuff type is important for BFRT. The cuffs used in BFRT can be categorized into inflatable and non-inflatable types. Inflatable cuffs include specialized elastic inflatable cuffs (Yasuda et al., 2012) and nylon inflatable cuffs (non-elastic) (Freitas et al., 2017), while non-inflatable cuffs consist of elastic types [e.g., elastic straps with buckles (Yamanaka et al., 2012) and elastic knee braces (Luebbers et al., 2014)] and non-elastic types [e.g., tourniquets (Shinohara et al., 1997)]. Different cuff types vary in material, width, and pressure quantifiability. Studies indicate comparable efficacy between elastic and nylon cuffs under matched width and pressure conditions (Loenneke et al., 2014), offering flexibility in material selection. However, pressure must be individualized to avoid excessive occlusion risks (Dankel et al., 2017).

Current guidelines recommend that pressure protocols should be individualized based on both cuff characteristics and anthropometric measurements (Patterson et al., 2019). Cuff width significantly influences the required occlusion pressure, with wider cuffs (e.g., 12 cm) necessitating lower pressures compared to narrower counterparts (e.g., 5 cm) (Jessee et al., 2016; Loenneke et al., 2012c). Additionally, larger limb circumscriptions require proportionally higher occlusion pressures (Loenneke et al., 2015a; Loenneke et al., 2012 J.).

To optimize safety and efficacy, cuff selection should be tailored to individual characteristics and training objectives. For athletes, narrower cuffs are recommended as they can reduce muscle growth inhibition beneath the cuff (Ellefsen et al., 2015; Jakobsgaard et al., 2018; Kacin and Strazar, 2011), thereby enhancing training outcomes and athletic performance (Wortman et al., 2021). Additionally, narrower cuffs help minimize discomfort during upper-limb training (Spitz et al., 2019; Fitzgibbons et al., 2012).

For elderly patients with sarcopenia, inflatable elastic cuffs are preferred due to their precise pressure control (Yasuda et al., 2012), reducing cardiovascular risks. The recommended cuff width is 5–9 cm for the upper limbs and 10–15 cm for the lower limbs to ensure even pressure distribution and effective blood flow restriction (Patterson et al., 2019; Mallmann et al., 2024). Close monitoring of adverse reactions such as pain and fatigue during compression is also advised (Wernbom et al., 2006; Rossow et al., 2012).

### 3.2 Restrictive pressure

In the early stages of BFRT research, studies commonly applied uniform cuff pressures without accounting for individual variations (Loenneke et al., 2013). These absolute pressures ranged from as low as 50 mmHg (Kubota et al., 2011) to as high as 300 mmHg (Cook et al., 2007). Although most studies demonstrated that using uniform absolute pressures could still produce beneficial muscular adaptations, higher BFR pressures may potentiate cardiovascular responses and are frequently accompanied by significant discomfort (Jessee et al., 2017; Mattocks et al., 2017).

Current guidelines emphasize that pressure settings should be based on both cuff specifications and individual participant characteristics (Patterson et al., 2019). As mentioned earlier, cuff width (Jessee et al., 2016; Loenneke et al., 2012c) and limb dimensions (Loenneke et al., 2015a; Loenneke et al., 2012 J.) significantly influence pressure setting. Additionally, gender (Jessee et al., 2016), limb dominance (Tafuna’i et al., 2021), and body position (Karanasios et al., 2022) represent important influencing factors. However, these effects are generally minimal in most individuals and often indistinguishable from measurement error. Theoretically, these variables can be accounted for through individualized arterial occlusion pressure (AOP) measurement, which is considered the gold standard for pressure prescription. AOP refers to the minimum pressure threshold required to completely restrict blood flow in a limb and can be determined directly using methods such as Doppler ultrasound or palpation of distal pulses (Loenneke et al., 2012b). This is done by applying a pressure cuff at the proximal limb (e.g., upper arm or thigh root), monitoring pulse signals at the distal artery (e.g., radial artery or dorsalis pedis artery) using a Doppler flowmeter, gradually increasing cuff pressure until complete disappearance of pulse signals, at which point the recorded pressure reading represents the individualized AOP value (Loenneke et al., 2025).

In addition to direct AOP measurement, alternative methods exist to quantify the degree of blood flow restriction or estimate occlusion pressure. The placement of devices such as pulse oximeters distal to the cuff can objectively (though indirectly) assess vascular occlusion by quantifying reductions in peripheral oxygen saturation (SpO_2_) levels, reflecting impaired arterial inflow and/or venous outflow (Beaume et al., 2025). Furthermore, near-infrared spectroscopy (NIRS) can be employed to measure oxygenation status in muscle tissue of the occluded limb, providing an indirect evaluation of both the degree and effectiveness of blood flow restriction (Simpson et al., 2025). When occlusion is effectively achieved, characteristic patterns typically include decreased tissue oxygenation index and elevated deoxygenated hemoglobin levels (Simpson et al., 2025).

Several studies have proposed pressure prescription methods based on a percentage of systolic blood pressure (SBP) (Brandner et al., 2015; Kim et al., 2017; Cook et al., 2007). For instance, in the study by Kim et al. (Kim et al., 2017), BFR pressure was set at 130% of individual SBP. Previous research supports this approach, demonstrating that 130% SBP effectively induces muscular adaptations and fatigue while maintaining safety (Cook et al., 2007). However, although this SBP-based method accounts for individual blood pressure variations, it still results in significant inter-individual differences in the degree of vascular occlusion. This approach is notably less precise than individualized AOP measurement (Younger et al., 2004).

For elastic bands where objective pressure quantification is unattainable, a subjective pressure rating of 7 out of 10 (with 10 indicating maximal tolerable pressure without discomfort or pain) may serve as the BFRT pressure standard (Wilson et al., 2013). Doppler ultrasound validation has confirmed that this 7/10 standard achieves venous occlusion without complete arterial flow blockade, thereby ensuring safety (Wilson et al., 2013). However, this method presents practical limitations due to inter-individual variability in pressure perception and its dependence on operator expertise.

Therefore, it is currently recommended to set BFR training pressure based on measured AOP, with the range of 40%–80% AOP being empirically supported (Patterson et al., 2019).

### 3.3 Intermittent and continuous BFR

In BFRT-related studies, the use of cuffs during exercise can be categorized into intermittent application (releasing restrictive pressure during rest intervals) and continuous application (maintaining restrictive pressure during rest intervals). However, whether restrictive pressure should be released during rest intervals remains controversial. Some studies support maintaining pressure during rest intervals to sustain BFR effects, while others suggest that releasing pressure can reduce discomfort and potential risks. Currently, most studies recommend maintaining pressure during rest intervals in BFRT (Hackney et al., 2012). Continuous application of restrictive pressure helps sustain BFR, increase cellular swelling in muscle cells, and positively impact interventions for sarcopenia patients (Naylor et al., 2005). Research indicates that continuous-pressure BFRT significantly enhances muscle protein synthesis rates and GH levels (Fujita et al., 2007). Yasuda et al. (2015) found that continuous-pressure BFRT during low-load training significantly increased muscle CSA and strength. However, compared to continuous pressure, intermittent BFR application reduces discomfort and improves safety. A randomized controlled trial by Schwiete et al. (2021) revealed significant differences in subjective experience and compliance between intermittent and continuous BFR. Intermittent BFR reported lower discomfort (VAS score 3.2/10) compared to continuous BFR (5.8/10, p < 0.05) and higher training compliance (92% vs. 85%). This difference may stem from the reduction in nerve compression and metabolite accumulation due to intermittent pressure release. Moreover, releasing pressure during rest intervals facilitates local blood flow recovery, potentially enhancing muscle recovery and adaptive responses (Abe et al., 2006).

Older adults with sarcopenia often suffer from multiple chronic conditions and functional decline, resulting in lower tolerance to BFRT. Based on these findings, intermittent BFR offers significant advantages in safety, tolerance, and muscle recovery, making it more suitable for older adults with sarcopenia.

### 3.4 Training load and intensity

Training load and intensity are critical considerations in BFRT interventions for sarcopenia. Commonly used intensity indicators in BFRT-related studies include percentage of 1RM, maximal voluntary contraction (MVC), and rating of perceived exertion (RPE). Among these, 1RM serves as the most frequently utilized intensity indicator.

A recent meta-analysis showed that BFRT had the greatest effect on muscle hypertrophy and strength when the intensity was 15%–30% 1RM/MVC (Loenneke et al., 2012d). For instance, Laurentino et al. (2012) demonstrated that 8 weeks of BFR combined with low-load (20% 1RM) knee extension training resulted in a 40.1% increase in maximal knee extension strength and a 6.3% increase in quadriceps CSA. Compared to traditional training at the same intensity, BFRT achieves significant improvements in muscle mass and strength at lower intensities, with an optimal range of 20%–40% 1RM (Lixandrão et al., 2015). However, these studies primarily focused on younger populations, limiting their applicability to older adults.

For older adults, Patterson and Ferguson, (2011) used a 25% 1RM load for BFRT and observed significant strength improvements and increased post-occlusive calf blood flow. Karabulut et al. (2010) found that low-intensity BFRT (20% 1RM) enhanced leg muscle strength to a level comparable to high-intensity resistance exercise (80% 1RM). Additionally, Vechin et al. (2015) confirmed that low-intensity BFRT (20%–30% 1RM) significantly promotes muscle strength and mass in older adults. Recently, Kong et al. (2023) conducted a systematic review and meta-analysis on the effects of BFRT on muscle strength and mass in older adults, with included studies utilizing training loads ranging from 20% to 50% 1RM and reporting no adverse events. However, higher training intensities may not always be beneficial. Studies suggest that higher intensities (e.g., 50% 1RM vs. 30% 1RM) may not result in significant differences in acute responses (Jessee et al., 2018) or muscle hypertrophy (Madarame et al., 2008). Furthermore, training intensities exceeding 40%–50% 1RM/MVC may cause cuff pressure to surpass SBP, leading to arterial occlusion (Yamada et al., 2004). Training under such conditions not only increases pain but may also pose safety risks (Loenneke et al., 2011b).

In summary, based on current evidence, a training load of 20%–40% 1RM or an RPE of 6–8 (on a 10-point scale) is recommended for older adults with sarcopenia (Yasuda et al., 2015). Low-load BFRT significantly enhances muscle protein synthesis, increases muscle mass and strength, and reduces joint load and injury risk.

### 3.5 Training frequency and inter-set rest intervals

For older adults with sarcopenia, training frequency and inter-set rest intervals are critical factors influencing training efficacy and recovery. However, there is no consensus on the optimal BFRT training frequency and rest intervals for this population. Generally, a training frequency of 2-3 sessions per week is sufficient to achieve significant improvements in muscle strength and mass (Kong et al., 2023). For example, Loenneke et al. (2012d) found that older women performing BFRT twice weekly experienced significant increases in knee extension strength and muscle thickness. However, some studies suggest that higher training frequencies (e.g., 4 sessions per week) may yield greater benefits. Clarkson et al. (2017) demonstrated that older men performing BFRT four times weekly showed superior improvements in muscle strength and function compared to a control group training twice weekly.

Since metabolic byproducts accumulated during training are partially cleared during rest intervals, the duration of inter-set rest plays a crucial role in modulating metabolic stress (de Freitas et al., 2017). Loenneke et al. (2015b) suggested that shorter rest intervals during BFRT increase metabolic stress, promoting muscle growth. However, for older adults with sarcopenia, excessively short rest intervals may lead to fatigue accumulation and increased injury risk. Studies indicate that rest intervals of 30–60 s are safe and effective for this population. For instance, Cook et al. (2018) found that a BFRT protocol with 30-s rest intervals significantly improved muscle strength and function in older women.

In summary, for older adults with sarcopenia, a BFRT frequency of 2-3 sessions per week with 30–60 s of inter-set rest is recommended. Excessive training frequency may hinder recovery, while prolonged rest intervals may reduce training efficiency.

### 3.6 Training volume

Training volume, comprising the number of sets and repetitions, is primarily influenced by training load, intensity, restriction pressure, and inter-set rest periods. For elderly patients with sarcopenia, determining the optimal number of sets and repetitions is vital to ensure training efficacy while avoiding the risks of overtraining.

Research indicates that 3-4 sets are safe and effective for elderly sarcopenia patients (Patterson and Ferguson, 2011). For instance, Abe et al. (2005) found that elderly men following a 3-set BFRT regimen showed significant improvements in muscle strength and thickness. However, some studies suggest that a higher number of sets may yield greater benefits. Cook et al. (2018) observed that elderly women on a 5-set BFRT regimen experienced superior improvements in muscle strength and functionality compared to those on a 3-set regimen. Regarding repetitions, a study demonstrated that a 15-repetition BFRT regimen significantly enhanced knee extension strength and muscle thickness in elderly women (Loenneke et al., 2012d). Conversely, Clarkson et al. (2017) found that elderly men on a 30-repetition BFRT regimen showed better improvements in muscle strength and functionality than those on a 15-repetition regimen. However, it is important to note that more repetitions are not always better, as excessively prolonging training duration or increasing repetitions can lead to overtraining. Additionally, beginners may struggle to meet the required repetition counts initially (Wernbom et al., 2012). Therefore, for elderly sarcopenia patients with decreased muscle mass, strength, and functionality, adjustments such as reducing exercise load, decreasing restriction pressure, and extending inter-set rest periods can help achieve the desired repetition counts. In conclusion, individuals new to BFRT should gradually adapt to the training stimulus before incrementally increasing training volume to ensure feasibility and safety.

In summary, significant progress has been made in the development of BFRT protocols. BFRT offers the unique advantage of achieving greater muscular adaptive responses at lower load intensities, making it particularly suitable for the rehabilitation of elderly patients with sarcopenia. When implementing BFRT, it is recommended to use inflatable cuffs with quantifiable pressure, with cuff widths of 5–9 cm for the upper limbs and 10–15 cm for the lower limbs. The restriction pressure should be individualized, typically set at 40%–80% AOP. An intermittent pressure mode should be adopted to avoid continuous compression, with inter-set rest periods of 30–60 s. The training load intensity should be 20%–40% of 1RM or controlled at 6-8 on the RPE scale (out of 10). The recommended training frequency is 2-3 sessions per week, with 3-4 sets per session and 15–30 repetitions per set. Additionally, BFR can be used alone or in combination with aerobic or resistance exercise. However, it is important to note that combining BFR with resistance exercise (Vechin et al., 2015) or aerobic exercise (Sugimoto et al., 2021) at the same intensity may lead to a more pronounced increase in heart rate and blood pressure. The following sections will provide a detailed discussion on the application modes of BFRT in the intervention of sarcopenia.

## 4 Application models of BFR in sarcopenia intervention

With the deepening understanding of the biological mechanisms and effects of BFRT, its application has gradually expanded from the field of sports and fitness to clinical rehabilitation, and its target population has shifted from healthy individuals and athletes to patients with clinical conditions. The primary characteristics of sarcopenia in the elderly include a significant decline in skeletal muscle strength (Cruz-Jentoft et al., 2019), as well as reduced stability and coordination of the musculoskeletal system (Lo et al., 2020). Studies have shown that BFR is applied during both voluntary resistance exercise (RE-BFR) (Chen et al., 2021; Parkington et al., 2022; Zhang et al., 2024) and aerobic exercise (AE-BFR) (Ozaki et al., 2011a; 2011b; Clarkson et al., 2017; Ferreira et al., 2017), and also passively without exercise (P-BFR) (Slysz et al., 2021; Fuchs et al., 2024). The multimodal application characteristics of BFRT provide clinicians with flexible protocol options. Rehabilitation professionals can precisely select appropriate intervention modalities based on patients’ functional status, rehabilitation goals, and clinical scenarios (Hackney et al., 2018; Valenzuela et al., 2018). The following section will systematically summarize the effects, relevant therapeutic parameters, and safety of BFR combined with aerobic exercise, resistance exercise, and standalone application, aiming to provide guidance for clinical practice.

### 4.1 AE-BFR

AE-BFR refers to a training method that involves the use of external pressure devices (such as tourniquets, elastic wraps, or inflatable cuffs) applied to the proximal limb during aerobic activities (e.g., walking, running, or cycling). This technique partially restricts venous return and reduces arterial blood flow, thereby promoting muscle hypertrophy, enhancing muscle strength, and improving cardiopulmonary function (Silva et al., 2019). This training method is highly adaptable and requires minimal equipment, making it suitable for most elderly patients with sarcopenia.

AE-BFR has been demonstrated to significantly enhance muscle strength and mass in older adults (De Lemos Muller et al., 2024). Clarkson et al. (Clarkson et al., 2017) found that BFR combined with walking training significantly improved knee extension strength and muscle thickness in elderly women. Ozaki et al. (2011b) further confirmed that after 10 weeks of BFR walking training, older participants experienced a 3.1% increase in thigh muscle CSA, a 3.7% increase in muscle volume, a 5.9% improvement in maximal isometric strength, and up to a 22% increase in isokinetic strength. Additionally, this training improved peak oxygen uptake (VO_2_peak) and carotid artery compliance, potentially reducing the risk of cerebrovascular events. Similar findings were reported in another study by Ozaki et al. (2011a). For elderly patients with osteoporosis, AE-BFR also demonstrated significant benefits, increasing the 1RM of knee extension (Pereira Neto et al., 2018). Moreover, AE-BFR positively impacted the cardiovascular system, enhancing cardiorespiratory endurance (Bennett and Slattery, 2019). Research indicates that BFR walking training not only significantly enhances VO_2_peak (Ozaki et al., 2011b) and VO_2_max (Thompson et al., 2024) but also improves carotid artery compliance in older adults (Ozaki et al., 2011a). Compared to high-intensity aerobic exercise (70% VO_2_max), low-intensity BFR walking training (40% VO_2_max) exerted less cardiovascular stress on older adults (Ferreira et al., 2017).

Regarding training parameters, AE-BFR typically involves exercise intensities of 40%–80% VO_2_max, 50%–70% maximum heart rate (HRmax), or 30%–45% heart rate reserve (HRR), with durations of 5–20 min, performed 2–3 times per week for 3–12 weeks. The occlusion pressure is usually set at 40%–80% AOP. This parameter configuration ensures training efficacy while minimizing cardiovascular risks. For patients with sarcopenia, it is recommended to start with lower pressures (40%–50% AOP) and shorter durations (5–10 min), gradually increasing intensity. Although AE-BFR has been demonstrated to be safe and effective in improving cardiovascular function while mitigating age-related physiological decline (Yasuda et al., 2015), special precautions must be taken when applying this intervention to elderly populations with cardiovascular diseases. Strict contraindication screening (including, but not limited to, abnormal D-dimer levels and history of active thrombosis) must be conducted prior to implementation. Continuous blood pressure and heart rate monitoring should also be maintained throughout the training session to ensure safety (Yasuda et al., 2017).

In conclusion, existing research indicates that AE-BFR promotes muscle hypertrophy, increases muscle strength, prevents muscle atrophy, enhances walking ability, and improves arterial compliance in older adults, while reducing post-exercise cardiovascular stress. However, further studies are needed to optimize training parameters and evaluate long-term efficacy and safety. Table 2 summarizes the effects of AE-BFR based on relevant studies.

**TABLE 2:** Effects of AE-BFR in older adults: summary of relevant studies.

Study population	Age (Years)	Study design	AE-BFR protocol	Outcomes	References
Sedentary Elderly Men and Women	60–80	Randomized Controlled Trial	Low-intensity BFR-walking, 4 sessions/week for 6 weeks, pressure at 60% LOP.	AE-BFR significantly improved physical function, particularly lower limb strength, aerobic capacity, and dynamic balance in elderly individuals	Clarkson et al. (2017)
Sedentary Women	57–73	Randomized Controlled Trial	20 min treadmill BFR-walking at 45% HRR, 4 days/week for 10 weeks, pressure at 160–200 mmHg	AE-BFR significantly improved thigh muscle volume, strength, functional capacity, and aerobic capacity in elderly women	Ozaki et al. (2011b)
Sedentary Men and Women	57–76	Randomized Controlled Trial	BFR-walking at 45% HRR for 20 min, 4 days/week for 10 weeks, occlusion pressure: 160–200 mmHg	AE-BFR significantly increased thigh muscle volume and strength, and improved carotid artery compliance	Ozaki et al. (2011a)
Female Osteoporosis Patients	61.40 ± 4.63	Randomized Controlled Trial	BFR low-intensity aerobic exercise at 65% HRmax for 15 min, 2 sessions/week for 12 weeks, occlusion pressure: 80% AOP.	AE-BFR significantly improved knee extension 1RM.	Pereira Neto et al. (2018)
Healthy young adults	26 ± 11	Randomized Controlled Trial	BFR treadmill walking protocol: High-frequency BFR group (twice daily for 4 weeks, 35 sessions total) vs. Low-frequency BFR group (twice weekly for 6 weeks, 12 sessions total)	Both high- and low-frequency BFR training significantly improved VO_2_max, though high-frequency training demonstrated faster adaptation	Thompson et al. (2024)
Healthy Older Adults	63.8 ± 4.2	Randomized Crossover Trial	BFR-walking at 40% VO_2_max for 20 min, occlusion pressure: 50% AOP.	Low-intensity AE-BFR demonstrated lower cardiovascular stress in post-exercise cardiac autonomic and hemodynamic responses	Ferreira et al. (2017)

### 4.2 RE-BFR

While aerobic exercise can enhance muscle strength, it requires long-term adherence to achieve significant results. In contrast, RE-BFR can significantly increase muscle strength and volume in the trained limbs within a shorter period (Lo et al., 2020). Among these, BFR combined with low-intensity resistance exercise (LRE-BFR) is particularly suitable for elderly patients with sarcopenia and those with limited mobility due to underlying conditions, as it involves lower loads and does not result in adverse effects such as prolonged muscle function decline, muscle soreness, or muscle damage (Hughes et al., 2017).

Research has shown that LRE-BFR can significantly increase muscle CSA and strength in elderly patients with sarcopenia. For example, a case report by Grutter Lopes et al. (2019) demonstrated that after 3 months of LRE-BFR (30% 1RM), an elderly patient with sarcopenia experienced a 17.9% increase in grip strength, a 4.6% increase in maximal isokinetic knee extension strength, and a 2.1% increase in limb skeletal muscle mass. Additionally, LRE-BFR yields comparable or even superior results to traditional high-intensity resistance exercise. Cook et al. (2017) found that both LRE-BFR (30%–50% 1RM) and high-intensity resistance exercise (70% 1RM) improved muscle CSA in elderly individuals, although the effects of high-intensity resistance exercise were more pronounced initially. However, after 12 weeks of training, the differences between the two approaches diminished. Karabulut et al. (2010) also confirmed this, showing that LRE-BFR (20% 1RM) was nearly as effective as high-intensity resistance exercise (80% 1RM) in increasing muscle strength in elderly men. Overall, LRE-BFR (20%–30% 1RM) can produce similar muscular adaptations as high-intensity resistance exercise (70%–80% 1RM) while reducing joint stress (Fabero-Garrido et al., 2022). Furthermore, studies have found no adverse events such as muscle function decline, severe soreness, or increased muscle damage in sarcopenia patients following LRE-BFR (Barbalho et al., 2019). These characteristics make LRE-BFR particularly suitable for elderly individuals and patients with sarcopenia, as it maximizes training benefits while minimizing injury risks. RE-BFR not only prevents sarcopenia but also treats muscle loss caused by other conditions, such as knee osteoarthritis. Ferraz et al. (2018) demonstrated that LRE-BFR (30% 1RM) increased muscle CSA and improved physical function (e.g., timed up-and-go test and sit-to-stand test) in elderly patients with knee osteoarthritis, with effects similar to those of high-intensity resistance exercise (80% 1RM).

Regarding training parameters, it is recommended to use 40%–80% LOP combined with low-load resistance exercise (20%–30% 1RM), performing 3-4 sets of 15–20 repetitions per session, with 30–60 s of rest between sets, 2–3 times per week (Chen et al., 2021). For patients with sarcopenia, it is advisable to start with lower pressure (40%–50% LOP) and fewer sets (2-3 sets), gradually increasing intensity (Zhang et al., 2024). Safety assessments indicate that BFRT may be an alternative to reduce muscle wasting in the elderly population, but it should be used with caution in patients with hypertension or impaired cardiovascular function (Scott et al., 2018). In addition, the possibility of pain and discomfort should still be noted (Parkington et al., 2022). Therefore, it is recommended to conduct a comprehensive musculoskeletal assessment before training and adhere to the principle of progressive overload (e.g., gradually increasing training intensity, repetitions, or occlusion pressure) (Lixandrão et al., 2018) during the training process, while closely monitoring subjective discomfort to ensure both safety and effectiveness of the training.

In summary, RE-BFR provides a safe and effective training modality for elderly individuals and patients with sarcopenia, enhancing muscle strength and improving physical function while reducing joint stress, with effects comparable to high-intensity resistance exercise. However, further research is needed to optimize training parameters and evaluate long-term efficacy and safety. Table 3 summarizes the findings from relevant studies on RE-BFR.

**TABLE 3:** Effects of RE-BFR in older adults: summary of relevant studies.

Study population	Age (Years)	Study design	RE-BFR protocol	Outcomes	References
Elderly Individuals	≥65	Randomized Controlled Trial	LRE-BFR (30%–50% 1RM) leg training, 2 sessions/week for 12 weeks, average pressure 184 ± 25 mmHg	After 12 weeks of training, LI-BFR demonstrated comparable improvements in muscle strength to high intensity restriction (HRE; 70% 1RM)	Cook et al. (2017)
Elderly Male with Sarcopenia	91	Case Study	Low-intensity training (30% 1RM) for 12 weeks, followed by 8 weeks of LRE-BFR (50% resting systolic pressure) after 1-month rest	LRE-BFR outperformed low-intensity training in improving muscle strength, muscle mass, and IGF-1 levels. LRE-BFR also positively affected vascular function and inflammatory markers but may increase oxidative stress	Grutter Lopes et al. (2019)
Healthy Middle-Aged and Elderly Men	50–64	Randomized Controlled Trial	Subjects performed upper extremity training 3 times per week and lower extremity training with (20% 1RM, 205.4 ± 4.3 mmHg) or without (80% 1RM) BFR 2 times per week for 6 weeks	LRE-BFR was as effective as HRE in increasing leg muscle strength in elderly men	Karabulut et al. (2010)
Comatose Elderly ICU Patients	66 ± 4.3	Randomized Controlled Trial	BFR passive activity (no load) on both lower limbs, once daily for the entire hospitalization period, pressure at 80% of anterior tibial artery systolic pressure	BFR combined with passive activity significantly reduced muscle atrophy in elderly ICU patients, with no adverse effects observed	Barbalho et al. (2019)
Female Patients with Knee Osteoarthritis	50–65	Randomized Controlled Trial	LRE-BFR (30% 1RM) for 12 weeks, average pressure of 97.4 ± 7.6 mmHg applied at the groin	LRE-BFR increased muscle strength, quadriceps mass, and function in knee osteoarthritis patients, with effects similar to HRE (80% 1RM). However, LRE-BFR imposed less stress on joints and was more effective in reducing pain	Ferraz et al. (2018)
Community-Dwelling Elderly with Sarcopenia	≥65	Randomized Controlled Trial	LRE-BFR (20%–30% 1RM), 3 sessions/week for 12 weeks, pressure at 50% LOP.	LRE-BFR and HRE (60%–70% 1RM) were similarly effective in increasing muscle strength, improving body composition, muscle function, pulmonary function, blood biomarkers, and cardiovascular risk factors	Chen et al. (2021)
Elderly with Sarcopenia	≥65	Randomized Controlled Trial	LRE-BFR (20%–30% 1RM), 3 sessions/week for 12 weeks, pressure at 50% LOP.	LRE-BFR significantly improved muscle strength, body composition, cardiovascular risk factors, and blood biomarkers	Zhang et al. (2024)
Elderly Women	63–75	Randomized Crossover Trial	LRE-BFR (20% 1RM), pressure set at 50% AOP, 3 sets with 30 s rest between sets, 8 min rest between exercises	LRE-BFR may cause acute increases in blood pressure, so patients with hypertension or cardiovascular diseases should use it with caution	Scott et al. (2018)
Healthy Elderly Individuals	64.3 ± 4.2	Randomized Crossover Trial	LRE-BFR (20% 1RM), pressure set at 50% AOP, 4 sets with 30 s rest between sets, 5 min rest between exercises	LRE-BFR elicits higher perceived pain and fatigue in older adults, yet still promotes positive affective responses post-exercise	Parkington et al. (2022)

### 4.3 P-BFR

During periods of illness, injury, surgery, joint immobilization, or prolonged bed rest, reduced weight-bearing activities lead to significant declines in muscle strength and mass, resulting in functional impairments (Parry and Puthucheary, 2015). Studies have shown that after 30 days of bed rest, quadriceps strength decreases by 20%–60% (Stevens et al., 2003), and the muscle mass of the knee extensors decreases by an average of 11%–16% (Cook et al., 2010). Therefore, for elderly patients with sarcopenia or those unable to perform early load-bearing exercises post-surgery, early rehabilitation interventions using P-BFR can be used to mitigate the loss of strength and muscle size due to discontinuation (Hughes et al., 2017).

The mechanism of BFR without muscle contraction resembles ischemic preconditioning, involving cycles of ischemia-reperfusion, with some researchers considering them functionally equivalent (Patterson et al., 2019). In a follow-up study involving healthy subjects with cast immobilization, Kubota et al. (2008) found that applying BFR for 14 days effectively prevented disuse-induced weakness in knee extensors and flexors and reduced declines in thigh and calf circumference. Another study on cast immobilization demonstrated that repeated BFR at 50 mmHg cuff pressure reduced chronic unloading-induced muscle weakness (Kubota et al., 2011). Additionally, Takarada et al. (2000) found that applying blood flow restriction (BFR) (200–260 mmHg, twice daily, with 5 min of inflation followed by 3 min of rest, repeated 5 times) significantly decreased quadriceps atrophy after anterior cruciate ligament (ACL) surgery (9.4% vs. 20.7%). These findings suggest BFR may suppress muscle atrophy by downregulating MuRF1 expression (Kakehi et al., 2020). However, two recent studies failed to observe BFR’s benefits against bed rest (Fuchs et al., 2024) or knee brace immobilization (Slysz et al., 2021).

Collectively, BFR alone may be insufficient to fully prevent muscle loss. Notably, methodological heterogeneity (e.g., immobilization protocols, pressure parameters) complicates comparisons. Although there may be differences in the progression of atrophy due to different braking modalities, some form of muscle contraction appears to be essential for maintaining skeletal muscle tissue (Cook et al., 2010). This contraction may not be active, as studies have found that electrical stimulation combined with BFR has equally protective effects (Slysz et al., 2021; Natsume et al., 2015). However Iversen et al. (2016) found no atrophy mitigation even with combined BFR and contraction. Moreover, special attention must be paid to population-specific protocols for P-BFR therapy in clinical practice. For instance, in orthopedic postoperative patients, the compression area should strictly avoid cast or external fixator sites, with implementation delayed for at least 72 h post-surgery to prevent active bleeding (Takarada et al., 2000). Table 4 summarizes BFR effects in the absence of exercise.

**TABLE 4:** Effects of P-BFR in adults: summary of relevant studies.

Study population	Age (Years)	Study design	BFR protocol		Outcomes	References
Healthy Males	Not Specified	Randomized Controlled Trial	The left ankle was immobilized with a cast, and BFR was applied at 200 mmHg for 5 min followed by 3 min of rest, repeated 5 times per session, twice daily for 14 days		Periodic BFR effectively prevented declines in muscle strength and reductions in muscle circumference during 2 weeks of immobilization and unloading, but did not significantly alter GH levels	Kubota et al. (2008)
Healthy Males	Mean Age: 22.8	Randomized Controlled Trial	The left ankle was immobilized with a cast, and BFR was applied at 50 mmHg for 5 min followed by 3 min of rest, repeated 5 times per session, twice daily for 14 days		Repeated BFR at low pressure (50 mmHg) partially alleviated muscle weakness induced by immobilization and unloading, but had limited effects on preventing muscle atrophy	Kubota et al. (2011)
Post-ACL Reconstruction Patients	20.0–23.3	Randomized Controlled Trial	BFR (180–238 mmHg) was applied twice daily starting from the 3rd postoperative day, with each session consisting of 5 min of occlusion followed by 3 min of release, repeated 5 times, for 14 days		BFR significantly reduced disuse atrophy in the knee extensors, but had no significant effect on the knee flexors	Takarada et al. (2000)
Healthy males	24 ± 3	Randomized Crossover Trial	BFR (200 mmHg) applied to one leg 3 times daily for 2 weeks during bed rest		BFR failed to regulate muscle protein synthesis or preserve muscle mass, strength, or oxidative capacity	Fuchs et al. (2024)
Healthy adults	22 ± 3	Randomized Controlled Trial	Unilateral limb unloading (knee brace + crutches) for 14 days	BFR (180–290 mmHg)	Neither BFR combined with electrical stimulation nor BFR alone preserved muscle strength	Slysz et al. (2021)
				BFR with electrical stimulation		
Healthy males	26.2 ± 0.7	Self-controlled Trial	Individualized BFR: 5 min pressure, 1 min rest, 4 cycles, twice daily, 5 days/week for 2 weeks		Low-intensity electrical stimulation combined with BFR induced muscle hypertrophy and strength gains, while stimulation alone was ineffective	Natsume et al. (2015)
			Electrical stimulation was continuously applied during BFR (8 s stimulation, 3 s rest, 30 Hz, 200 μs, intensity 5%–10% MVC)			
Post-ACL reconstruction athletes	18–40	Randomized Controlled Trial	BFR: 5 min pressure (130–180 mmHg), 3 min rest, 5 cycles, 14 days		Intermittent BFR did not significantly reduce quadriceps atrophy in athletes post-ACL reconstruction	Iversen et al. (2016)

In summary, comparisons between different exercise modalities indicate that RE-BFR is superior to AE-BFR in promoting muscle hypertrophy, while AE-BFR offers greater advantages in improving cardiorespiratory function (Centner et al., 2019). Additionally, P-BFR is more suitable for individuals who are bedridden or unable to perform load-bearing exercises. For elderly patients with sarcopenia, it is recommended to select the appropriate exercise modality based on individual conditions to achieve comprehensive improvements in muscle strength and cardiorespiratory fitness.

## 5 Safety and risks of BFRT intervention in sarcopenia

### 5.1 Safety

In the elderly population, BFRT has emerged as an effective intervention for treating age-related sarcopenia. This method not only promotes muscle strength but also reduces the risks associated with high-intensity training. Additionally, BFRT is cost-effective and easy to implement (Bahamondes-Ávila et al., 2020). However, in clinical applications, besides considering the benefits of BFRT, it is essential to prioritize its safety and potential adverse effects in elderly individuals with sarcopenia.

Clinical safety studies indicate that the incidence of adverse reactions to BFRT is low. A meta-analysis by Patterson et al. (2019) involving 47 studies reported that the incidence of BFRT-related adverse events was only 0.05%, primarily manifesting as transient limb numbness and other mild symptoms. A nationwide survey by Yasuda et al. (2017) further confirmed the safety of BFRT, revealing no serious risks such as paralysis due to nerve compression, pulmonary embolism, venous thrombosis, rhabdomyolysis, cerebral infarction, cerebral hemorrhage, or pulmonary infarction. Based on these findings, BFRT is considered a relatively safe intervention, although it should be conducted under professional supervision to minimize potential adverse effects (Bilski et al., 2022).

Moreover, BFRT does not negatively affect arterial stiffness. Yasuda et al. (2015) found that in healthy elderly individuals, hemodynamic parameters (e.g., heart rate and blood pressure), arterial stiffness index, vascular endothelial function, coagulation factors (fibrin degradation products [FDP] and D-dimer), and muscle damage markers (creatine kinase levels) showed no significant changes before and after BFRT, and no cardiovascular adverse events or muscle damage were observed. Ozaki et al. (2013) also reported that BFRT had no significant impact on arterial stiffness.

BFRT can also improve vascular endothelial function and peripheral circulation in healthy elderly individuals. Shimizu et al. (2016) demonstrated that after 4 weeks of LRE-BFR (20% 1RM), vascular endothelial growth factor and reactive hyperemia index significantly increased (both P < 0.01), while von Willebrand factor significantly decreased (P < 0.05), indicating improved vascular endothelial function.

Specialized studies on the elderly population further validate the safety of BFRT. Karabulut et al. (2013) found that 6 weeks of LRE-BFR (20% 1RM) did not increase resting inflammatory markers (IL-6) or muscle damage markers (CK). Additionally, BFRT has demonstrated dual benefits in chronic pain and rehabilitation populations, effectively alleviating pain while maintaining high safety (Song et al., 2021). For example, BFRT significantly improved pain, function, and joint mobility in patients with knee osteoarthritis, outperforming traditional resistance exercise with good safety (Bryk et al., 2016).

In summary, BFRT exhibits high safety and minimal adverse effects in cardiovascular, musculoskeletal, and special populations, making it a safe intervention for the elderly. However, individualized assessment and monitoring remain crucial, particularly for patients with severe cardiovascular diseases or bleeding tendencies. Table 5 summarizes relevant studies on the safety of BFRT.

**TABLE 5:** Safety of BFRT: summary of relevant studies.

Study population	Age (Years)	Study design	BFRT protocol	Safety report	References
Healthy Elderly	71 ± 4	Randomized Controlled Trial	Lower limb training combined with BFR (20% 1RM), 3 days per week, once daily, for 4 weeks. Cuff pressure set based on SBP.	BFRT improved vascular endothelial function and peripheral circulation in healthy elderly	Shimizu et al. (2016)
Healthy Elderly	61–85	Randomized Controlled Trial	LRE-BFR using elastic bands (26.7%–30.8% MVC), twice weekly, 75 repetitions per set, for 12 weeks. Cuff pressure: 120–270 mmHg	BFRT did not negatively affect arterial stiffness in the elderly	Yasuda et al. (2015)
Japanese KAATSU Training Association Members and Facility Users	20–60	Nationwide Questionnaire Survey	Individualized BFRT.	No serious adverse effects, such as cerebral hemorrhage, infarction, thrombosis, or rhabdomyolysis, were reported under scientific BFRT guidance	Yasuda et al. (2017)
Young Males	22–32	Randomized Controlled Trial	30% 1RM bench press training, 3 times per week, 30 repetitions followed by 3 sets of 15 repetitions with 30-s rest intervals, for 6 weeks	BFRT did not negatively affect carotid artery compliance	Ozaki et al. (2013)
Healthy Elderly Males	56.6 ± 0.6	Randomized Controlled Trial	Upper limb training (80% 1RM) 3 times per week and lower limb training with (20% 1RM) or without (80% 1RM) BFR, 3 days per week, for 6 weeks. Cuff pressure adjusted based on ability to complete all repetitions and RPE.	BFRT did not induce adverse inflammatory responses or increase muscle damage markers, demonstrating good safety and tolerability	Karabulut et al. (2013)
Females with Knee Osteoarthritis	Mean Age: 61	Randomized Controlled Trial	30% 1RM quadriceps training, 3 times per week, for 6 weeks (18 sessions total). Cuff pressure: 200 mmHg	Significant reduction in anterior knee discomfort during BFRT.	Bryk et al. (2016)

### 5.2 Risks

BFRT offers certain advantages in the intervention of sarcopenia in the elderly, but its potential risks cannot be overlooked, and safety concerns require careful attention (Manini and Clark, 2009). The primary effects of BFRT on limbs are the reduction of arterial blood flow and the accumulation of venous blood, leading to a state of relative ischemia and hypoxia (Yasuda et al., 2010). During this process, metabolic byproducts such as lactic acid cannot be effectively cleared (Yasuda et al., 2015), resulting in a significant increase in metabolic stress. Current research on the safety of BFRT primarily focuses on thrombosis, blood pressure elevation, microvascular dysfunction, endothelial cell apoptosis and damage, and neuromuscular dysfunction.

Thrombosis is a key area of safety research in BFRT. Nakajima et al. (Nakajima et al., 2007) were the first to systematically evaluate the effects of BFRT on hemostatic function, finding no significant changes in thrombosis markers (e.g., D-dimer and FDP) after training, while tissue plasminogen activator (tPA) significantly increased (a decrease in tPA may indicate a risk of thrombosis). Although oxygen saturation decreased in a low-pressure chamber, the study found no serious adverse effects related to hypoxia, providing preliminary evidence for the safety of BFRT. Subsequently, Zaar et al. (2009) further elucidated the mechanism of external pressure on the coagulation system through simulated negative pressure environments. Although an increase in thrombin generation markers such as thrombin-antithrombin III complex (TAT) was observed, the levels were far below pathological thresholds, and D-dimer levels remained unchanged, indicating no stable thrombus formation. This early activation of the coagulation system may be related to physiological responses in mild to moderate blood loss states. Building on this, Madarame et al. (2010) specifically studied LRE-BFR and found that it did not significantly increase thrombin generation markers (e.g., prothrombin fragment 1 + 2 [PTF] and TAT)) or intravascular clot formation markers (e.g., D-dimer and FDP), indicating no significant thrombosis risk under these training conditions. Although BFRT led to a significant decrease in plasma volume, no activation of the coagulation system was observed, further supporting the safety of BFRT. These studies suggest that scientifically applied BFRT does not induce thrombosis, but further research is needed to assess thrombosis risks in postoperative patients undergoing BFRT rehabilitation. Studies by Bond et al. (2019) and Hughes et al. (2019) showed that even in orthopedic postoperative patients, BFRT did not increase the risk of venous thromboembolism, with safety profiles comparable to those of healthy populations. Additionally, long-term safety studies (Cook et al., 2017; Neto et al., 2017) further support this conclusion, indicating that BFRT interventions lasting more than 12 weeks do not cumulatively increase thrombosis risk. Although these studies differ in methods and populations, they consistently conclude that BFRT does not significantly increase thrombosis risk. However, caution is still advised when using BFRT in elderly individuals with cardiovascular diseases or other health issues (Centner et al., 2019).

Recent studies have demonstrated that BFRT induces acute blood pressure elevation, primarily attributed to the accumulation of metabolic byproducts (Yamada et al., 2025). Compared to equivalent training without BFR, BFRT elicits more pronounced blood pressure fluctuations (Brandner et al., 2015; Downs et al., 2014). However, these hemodynamic changes remain within normal exercise-induced physiological ranges, with blood pressure returning to baseline levels within 5–10 min post-exercise (Rossow et al., 2012). The current evidence regarding the effects of repetitive BFRT on resting blood pressure remains insufficient (Wong et al., 2022). Notably, a recent large-scale study found no observable changes in resting blood pressure following repeated BFRT interventions (Spitz et al., 2024).

After BFRT, the reperfusion process may lead to cellular damage, manifesting as microvascular dysfunction, endothelial cell apoptosis and damage, and neuromuscular dysfunction. During reperfusion following BFRT, skeletal muscles acutely release inflammatory molecules and reactive oxygen species, impairing microvascular function (Hughes et al., 2017). Additionally, repeated reperfusion injury can affect endothelial function, increasing membrane permeability and generating reactive oxygen species, leading to endothelial cell damage (Madarame et al., 2013). In a study by Jenkins et al. (2013), BFRT (220 mmHg) was applied to 10 young males, revealing a significant increase in CD62E^+^ endothelial microparticles at 10 and 20 min (P < 0.05), indicating vascular endothelial activation. Simultaneously, CD31^+^/CD42b^−^ endothelial microparticles significantly increased (P < 0.05), suggesting endothelial cell apoptosis. This study demonstrates that excessive BFRT pressure can cause vascular endothelial activation, apoptosis, and damage in local tissues. Therefore, Da Cunha Nascimento et al. (2020) recommend setting the vascular occlusion pressure to 40%–60% AOP in practical use, achieving transient ischemia and hypoxia in local muscle groups while avoiding endothelial damage due to excessive pressure. Furthermore, ischemia-reperfusion may cause neuromuscular dysfunction, but related studies have only been conducted in mouse models, requiring further validation for human applicability (Wilson et al., 2019).

In conclusion, BFRT may pose potential risks to the human body, necessitating greater attention to safety during its application. However, given the limited number of high-quality clinical studies, small sample sizes, and lack of homogeneity in study populations—particularly the scarcity of reports on safety and adverse effects in elderly sarcopenia patients—further validation of these findings is required. Table 6 summarizes relevant studies on the potential risks of BFRT.

**TABLE 6:** Potential risks of BFRT: summary of relevant studies.

Study population	Age (Years)	Study design	BFRT protocol	Potential risks	Risk report	References
Healthy young males	23 ± 3	Randomized Crossover Trial	Unilateral bicep curls (20% 1-RM) with: Continuous low-pressure BFR (80% SBP, maintained throughout); Intermittent high-pressure BFR (130% SBP, released during rest intervals)	Blood pressure elevation	blood pressure returned to baseline within 5–10 min post-BFRT with no lasting effects	Rossow et al. (2012)
Healthy Males	48 ± 5	Self-controlled Trial	Subjects underwent 15 min of BFR (160 mmHg) in a low-pressure chamber, followed by approximately 10 min of low-intensity lower limb and foot aerobic exercise	Thrombosis, Hypoxia	Under low pressure and bed rest conditions, BFRT did not activate the coagulation cascade or induce thrombosis	Nakajima et al. (2007)
	30 ± 4	Randomized Crossover Trial	Subjects performed leg press exercises (30% 1RM) after 24 h of bed rest, with and without BFR (160 mmHg)			
Healthy Males	26 ± 3	Self-controlled Trial	Subjects underwent a 10-min lower body negative pressure test at 30 mmHg, lasting 10 min	Coagulation System Activation, Cardiovascular Response	BFRT significantly increased TAT levels, but D-dimer levels remained unchanged, indicating no stable thrombus formation	Zaar et al. (2009)
Healthy Adults	25.1 ± 2.8	Randomized Crossover Trial	Subjects performed low-intensity leg press training with and without BFR (150–160 mmHg)	Thrombosis, Cardiovascular Response	LRE-BFR did not activate the coagulation system or induce intravascular clot formation in healthy subjects	Madarame et al. (2010)
Patients with unilateral ACL reconstruction	Mean Age: 29	Randomized Controlled Trial	BFR combined with leg press (30% 1RM), twice weekly for 8 weeks. Average pressure: 150–157 mmHg	Discomfort, Pain	Some patients reported discomfort and pain, but no serious adverse events occurred	Hughes et al. (2019)
Patients with Ischemic Heart Disease (IHD)	57 ± 6	Randomized Controlled Trial	BFR combined with knee extension exercises (20% 1RM)	Endothelial Cell Damage	BFRT increased membrane permeability and reactive oxygen species production, leading to endothelial cell damage	Madarame et al. (2013)
Healthy Males	Mean Age: 29	Self-controlled Trial	220 mmHg pressure applied to the distal forearm and 40 mmHg to the proximal forearm to simulate blood flow disturbance for 20 min	Endothelial Activation, Apoptosis, and Damage	Disturbed blood flow significantly increased markers of endothelial activation and apoptosis (CD62E+ and CD31+/CD42b- endothelial microparticles)	Jenkins et al. (2013)
Male Mice	9–12 Weeks	Randomized Controlled Trial	Mice underwent voluntary running training for 5 weeks. Rubber tourniquets were used to induce hindlimb ischemia by applying them above the femoral greater trochanter for 1 h	Neuromuscular Dysfunction	BFRT poses a risk of ischemia-reperfusion injury leading to muscle and nerve dysfunction	Wilson et al. (2019)

## 6 Conclusions and future perspectives

BFRT presents a promising and innovative approach for counteracting sarcopenia in older adults. Current evidence supports its efficacy in enhancing muscle strength and function, underpinned by multifaceted mechanisms including anabolic hormone release, mTOR pathway activation, cellular swelling, and preferential type II fiber recruitment. For clinical implementation, individualized pressure prescription using appropriate cuff widths and low-load exercise intensities is paramount. BFR demonstrates versatility, being effective when combined with resistance or aerobic exercise, and shows potential benefit even in passive applications for immobilized individuals. While generally safe under proper guidance, vigilance for potential vascular, neurological, or muscular adverse effects is warranted, especially given the paucity of safety data specific to sarcopenic populations.

Despite providing practical recommendations based on current literature, key knowledge gaps persist. Future research must prioritize elucidating the precise biological mechanisms in the aging muscle, establishing optimal, individualized protocols, and expanding evidence within diverse sarcopenic cohorts, including those with varied etiologies beyond primary age-related loss. Crucially, the long-term functional outcomes and potential physiological trade-offs associated with BFRT in this vulnerable population require rigorous investigation to fully realize its therapeutic potential for improving mobility and quality of life (Loenneke et al., 2025).

## Abbreviations

4E-BP1, eukaryotic initiation factor 4E-binding protein 1; ACL, Anterior Cruciate Ligament; ActRIIB, activin receptor type-2B; AE-BFR, blood flow restriction combined with aerobic exercise; Akt, protein kinase B; AOP, arterial occlusion pressure; BFR, blood flow restriction; BFRT, blood flow restriction training; CSA, cross-sectional areas; FDP, fibrin degradation products; HRE, high-intensity restriction; HSP-70, heat shock protein-70; HSP-72, heat shock protein-72; HSPs, heat shock proteins; HRmax, maximum heart rate; HRR, heart rate reserve; IGF-1, insulin-like growth factor-1; IGF-1R, insulin-like growth factor 1 receptor; IRS-1, insulin receptor substrate-1; LOP, limb occlusion pressure; LRE-BFR, blood flow restriction combined with low-intensity resistance exercise; mTOR, mammalian target of rapamycin; MVC, maximal voluntary contraction; NO, nitric oxide; NOS, nitric oxide synthase; NOS-1, nitric oxide synthase-1; P-BFR, blood flow restriction (without exercise); PI3K, phosphatidylinositol 3-kinase; RE-BFR, blood flow restriction combined with resistance exercise; RPE, rating of perceived exertion; S6K1, ribosomal protein S6 kinase 1; SBP, resting systolic blood pressure; TAT, thrombin-antithrombin III complex; VO_2_max, maximal oxygen uptake; VO_2_peak, peak oxygen uptake.
